# Relapse in patients with limited‐stage ocular adnexal lymphoma treated by chemoimmunotherapy: Extended follow‐up of a phase 2 study

**DOI:** 10.1002/cam4.4639

**Published:** 2022-03-11

**Authors:** Sung‐Yong Kim, Won‐Sik Lee, Sung Yong Oh, Deok‐Hwan Yang, Hyo Jung Kim, Seong Kyu Park, Jae Wook Yang, Suk‐Woo Yang, Seok‐Goo Cho

**Affiliations:** ^1^ Department of Hematology, Konkuk University Medical Center Konkuk University School of Medicine Seoul Republic of Korea; ^2^ Department of Hematology and Oncology, Busan Paik Hospital Inje University College of Medicine Busan Republic of Korea; ^3^ Department of Internal Medicine Dong‐A University College of Medicine Busan Republic of Korea; ^4^ Department of Hematology‐Oncology Chonnam National University Hwasun Hospital, Chonnam National University Jeollanam‐do Republic of Korea; ^5^ Department of Internal Medicine Hallym University Sacred Heart Hospital, Hallym University College of Medicine Gyeonggi‐do Republic of Korea; ^6^ Department of Internal Medicine Soonchunhyang University Bucheon Hospital, Soonchunhyang University Gyeonggi‐do Republic of Korea; ^7^ Department of Ophthalmology, Busan Paik Hospital Inje University Busan Republic of Korea; ^8^ Department of Ophthalmology, Seoul St. Mary's Hospital The Catholic University of Korea Seoul Republic of Korea; ^9^ Division of Hematology, Catholic Blood and Marrow Transplantation Center, Seoul St. Mary's Hospital The Catholic University of Korea Seoul South Korea

**Keywords:** chemoimmunotherapy, mucosa‐associated lymphoid tissue lymphoma, ocular adnexal lymphoma, rituximab

## Abstract

**Background:**

Approximately 50% of limited‐stage ocular adnexal mucosa‐associated lymphoid tissue lymphoma (OAML) patients with adverse prognostic factors relapse after radiotherapy. Chemoimmunotherapy has been proposed as an alternative frontline therapy. However, only a few studies have reported its long‐term treatment outcome.

**Methods:**

In 2011, we commenced a phase 2 trial to investigate the efficacy of rituximab, cyclophosphamide, doxorubicin, and prednisolone (R‐CVP) in bilateral and non‐conjunctival limited‐stage OAML patients. Results of the clinical trial showed a response rate of 100% and a 4‐year progression‐free survival of 90.3% without significant toxicity. We extended the study period to December 2020 to determine the long‐term efficacy of R‐CVP chemoimmunotherapy.

**Results:**

At a median observation period of 66.0 months, eight of 33 study patients had relapsed. The cumulative incidence of relapse was 18.9% at 5 years and 44.7% at 8 years. The majority of relapses developed more than 4 years after treatment. Local relapse was more prevalent than distant relapse. The relapse risk of orbital and lacrimal diseases was likely to be higher than that of conjunctival and eyelid diseases (HR: 2.5, 95% CI: 0.498–12.500, *p* = 0.25).

**Conclusion:**

Although the response rate was remarkable for chemoimmunotherapy, the risk of late relapse was considerable. Based on our findings, clinical trials for limited‐stage OAML patients should have a long‐term observation period. To minimize radiation toxicity and reduce the risk of delayed relapse (local relapse and distant relapse), a future study with sequential or combination treatment of local low‐dose radiation and systemic chemoimmunotherapy can be considered.

## Graphical abstract

Although the response rate of the R‐CVP chemoimmunotherapy for the treatment of high risk, limited‐stage OAML was remarkable, the risk of late or delayed relapse was considerable. Clinical studies using chemoimmunotherapy should have a period of more than 5 years.

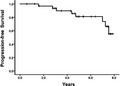

## 1 INTRODUCTION

Ocular adnexal mucosa‐associated lymphoid tissue lymphoma (OAML) is a type of extra‐nodal marginal zone lymphoma (EMZL) with an indolent course and a good response to radiotherapy. Therefore, radiotherapy has generally been recommended as a treatment of choice for patients with limited‐stage of this disease.^1^, ^2^, ^3^ However, a relapse rate of up to 25% has been reported in limited‐stage OAML,^4^ predominately in nonirradiated areas.^5^, ^6^, ^7^, ^8^, ^9^, ^10^ The relapse rate is substantial if the disease is bilateral or nonconjunctival.^5^, ^10^, ^11^, ^12^, ^13^ Theoretically, chemoimmunotherapy might lead to lower rates of distant relapse than radiotherapy.^14^, ^15^, ^16^ Therefore, chemoimmunotherapy might be a better frontline therapy option for patients with adverse prognostic factors than radiotherapy.

Radiation is a good treatment option for limited‐stage OAML. However, eyes are vulnerable to radiation toxicity. According to a previous report, a substantial portion of patients with OAML who received radiation therapy experienced various toxicities of radiation, including dry eye (59%), cataract ≥grade 2 (22%), and adnexal inflammation (25%) such as keratitis, blepharitis, and conjunctivitis.^17^ Therefore, for patients with OAML in both eyes, radiation therapy is not an easy option. In addition, patients with nonconjunctival disease have a higher risk of radiation complications than those with conjunctival disease because radiation therapy for nonconjunctival disease cannot be performed with a lens shield to protect the lens.^18^

Systemic chemoimmunotherapy has been attempted in a trial to avoid the following two disadvantages of radiotherapy: high risk of distant relapse and radiation complications. As for systemic treatment of EZML, it has been reported that a combination of rituximab and chemotherapeutic agents is superior in terms of treatment response to either rituximab monotherapy or chemotherapy.^14^, ^15^ However, long‐term outcomes were not reported in these previous studies. Data on chemoimmunotherapy for treating limited‐stage EZML are very scarce, especially for OAML. It remains unclear which regimen should be selected as the first‐line treatment for this category of disease.

In 2011, we conducted a phase 2 study to evaluate the efficacy of rituximab in combination with cyclophosphamide, vincristine, and prednisolone (R‐CVP)^19^ as a frontline therapy for newly diagnosed, limited‐stage, bilateral, or non‐conjunctival OAML patients (www.clinicaltrials.gov; identifier NCT01427114).^20^ Results of the clinical trial demonstrated the efficacy of R‐CVP with a response rate of 100% (complete response rate, 93.9%) at 2 years and a progression‐free survival of 90.3 ± 5.3% at 4 years. The study duration was 5 years. The median follow‐up duration for study patients was 50.6 months. However, many prior trials have revealed that EMZL has a tendency to relapse over time despite an initial good response to chemoimmunotherapy or radiotherapy.^11^, ^15^, ^18^, ^21^, ^22^ Thus, we extended that study with a longer follow‐up to evaluate the long‐term efficacy of this regimen. Here, we report a 9‐year analysis of the outcome of newly diagnosed, limited‐stage, bilateral, or non‐conjunctival OAML patients treated with R‐CVP.

## 2 SUBJECTS AND METHODS

### 2.1 Study design

This was an extended observational study of a single‐arm, multicenter, phase 2 clinical trial. The trial was commenced in 2011. It was extended to December 2020. Enrollment criteria were as follows: bilateral or non‐conjunctival Ann Arbor stage I–II OAM, which was bT1 or T >1, N0, and M0 by the tumor–node–metastasis (TNM) staging system for ocular adnexal lymphoma.^23^ Patients were treated with six cycles of R‐CVP every 21 days followed by two cycles of rituximab (375 mg/m^2^). This study was approved by the Institutional Review Boards of all participating centers. All patients provided written informed consent.

### 2.2 Study objective and assessments

The objective of this study was to explore the cumulative incidence of progression and progression‐free survival (PFS) over time. To evaluate progression or relapse, a contrast‐enhanced magnetic resonance imaging, an ophthalmic examination, and a full physical examination were repeated every 3 months during the first 2 years after treatment and every 6 months thereafter. When relapse or progression was suspected, a whole‐body computerized tomography and a positron emission tomography scan were taken to evaluate the presence of a distant relapse.

### 2.3 Statistical considerations

Progression‐free survival was defined as the time from initiation of treatment to lymphoma progression or death of any cause. Overall survival (OS) was defined as the time from initiation of treatment to death of any cause and censored at the time of the last visit.^24^ Time to complete response (CR) was defined as the time from treatment initiation to documented CR. Cox regression analysis was used for multivariable analysis of survival outcomes and disease progression. Prognostic factors considered in the analysis included gender, age, location of disease, TNM stage, elevated lactate dehydrogenase (LDH), and time to CR. All variables were included in multivariate analyses regardless of their significance in the univariate analysis. Continuous variables were categorized into dichotomous variables using a median split when multivariate analysis was conducted. *p* values were two‐sided. *p* < 0.05 was considered significant. All statistical analyses were performed using Statistical Package for Social Sciences, version 17.0 (SPSS).

## 3 RESULTS

### 3.1 Patient characteristics

A total of 33 patients were enrolled in this study. All patients completed the planned treatment without significant toxicity. Patients and disease characteristics are depicted in Table 1. The median age of patients was 49 years (range, 19–74 years). All study patients had performance scores of ECOG 0–1. No patient had B symptoms. Except for three patients who had elevated LDH levels, all other patients in this study had normal LDH levels.

**TABLE 1:** Patients and disease characteristics

Characteristics	Number (%)
Total	33
Gender	
Male	21 (64%)
Female	12 (36%)
Age, median (range)	49 years (19–74)
Disease location	
Orbit	13 (39.4%)
Conjunctiva	12 (36.4%)
Eyelid	5 (15.2%)
Lacrimal gland or duct	3 (9.1%)
TNM stage^a^	
T1N0M0	0 (0%)
bT1N0M0	10 (30.3%)
T2N0M0	16 (48.5%)
bT2N0M0	3 (9.1%)
T3N0M0	2 (6.1%)
bT3N0M0	1 (3%)
T4N0M0	1 (3%)
bT4N0M0	0 (0%)

Abbreviation: TNM, tumor, node, and metastasis.

^a^ TNM stage was based on the clinical staging system of ocular adnexal lymphoma proposed by the American Joint Committee on Cancer.

Anatomic locations of the disease included the conjunctiva in 12 (36.4%) patients, the orbit in 13 (39.4%), the eyelid in five (15.2%), and the lacrimal apparatus in three (9.1%). Fourteen (42.4%) patients had bilateral disease at presentation. All patients had stage IE disease based on the Ann Arbor staging. Based on TNM staging,^23^ 10 (30.3%) patients had bilateral T1 disease. The remaining 23 (69.7%) patients had T2 or higher disease.

### 3.2 Response

All study patients responded to the treatment (Table 2). At a median follow‐up of 66 months (range, 7.4–94.5 months), 32 (97.0%) patients achieved CR and one (3.0%) patient with disease originating from the eyelid had a partial response (PR) without progression until the last visit. The median time to CR in patients was 4.3 months (range, 1.8–64.1 months).

**TABLE 2:** Response to R‐CVP in limited‐stage ocular adnexal MALT lymphoma patients with bilateral or extra‐conjunctival involvement

	1 month after the completion of treatment	Best response during follow‐up	Last follow‐up
CR	28 (84.8%)	32 (97.0%)	24 (72.7%)
PR	5 (15.2%)	1 (3.0%)	1 (3.0%)
NR	0 (0%)	0 (0%)	0 (0%)
Progression	0 (0%)	0 (0%)	8 (24.2%)

Abbreviations: CR, complete response; IPI, international prognostic index; LDH, lactic dehydrogenase; NR, no response; PR, partial response.

### 3.3 Relapse and survival

Eight patients had a relapse (Table 3), including 2 (16.8%) of 12 patients with conjunctival disease, 0 (0%) of five patients with eyelid disease, four (30.8%) of 13 patients with orbit disease, and two (66.7%) of three patients with lacrimal apparatus disease. Notably, relapse in patients with lacrimal disease occurred at a different site from that of the primary tumor, whereas relapse in patients with disease at another location occurred at the same site as the primary tumor.

**TABLE 3:** Characteristics of relapsed patients

Patient	Location of primary disease	TNM	Relapse location (difference from the primary site)	Time to relapse (years)
Patient 3	Orbit	T4N0M0	Orbit (same)	7.0
Patient 7	Orbit	T2N0M0	Orbit (same)	7.5
Patient 10	Conjunctivae	bT1N0M0	Conjunctiva (same)	1.6
Patient 20	Lacrimal gland	bT2N0M0	Systemic (different)	4.4
Patient 23	Lacrimal sac	T2N0M0	Conjunctiva (different)	3.1
Patient 26	Orbit	bT2N0M0	Orbit (same)	7.2
Patient 27	Conjunctivae	bT1N0M0	Conjunctiva (same)	4.7
Patient 28	Orbit	T2N0M0	Orbit (same)	2.8

Abbreviation: TNM, tumor, node, and metastasis.

**FIGURE 1:**
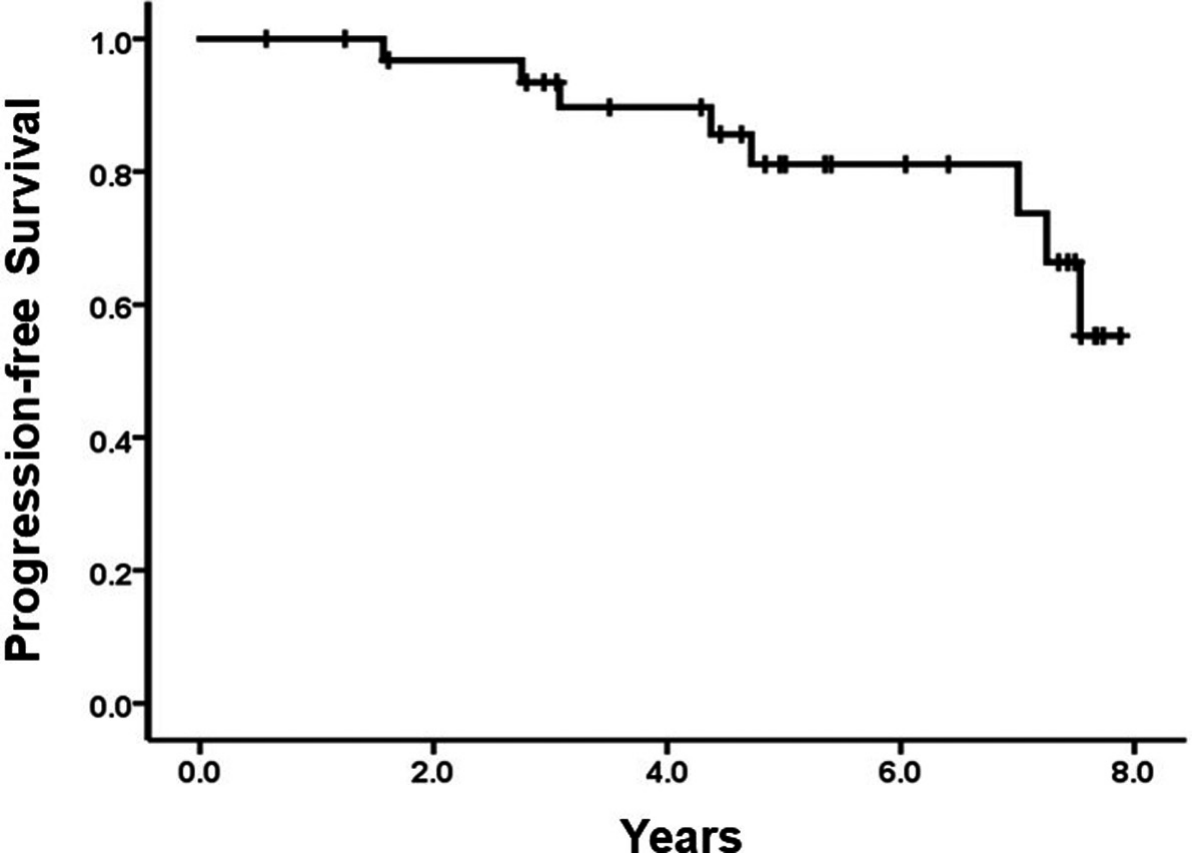
Progression‐free survival in patients with limited‐stage OAML having nonconjunctival or bilateral involvement

The median time to progression was 4.5 years (range, 1.6–7.5 years). The cumulative incidence of progression was 18.9% at 5 years and 44.7% at 8 years. The PFS was 81.1% at 5 years and 55.3% at 8 years (Figure 1). No death was observed. Thus, OS was 100%.

**FIGURE 2:**
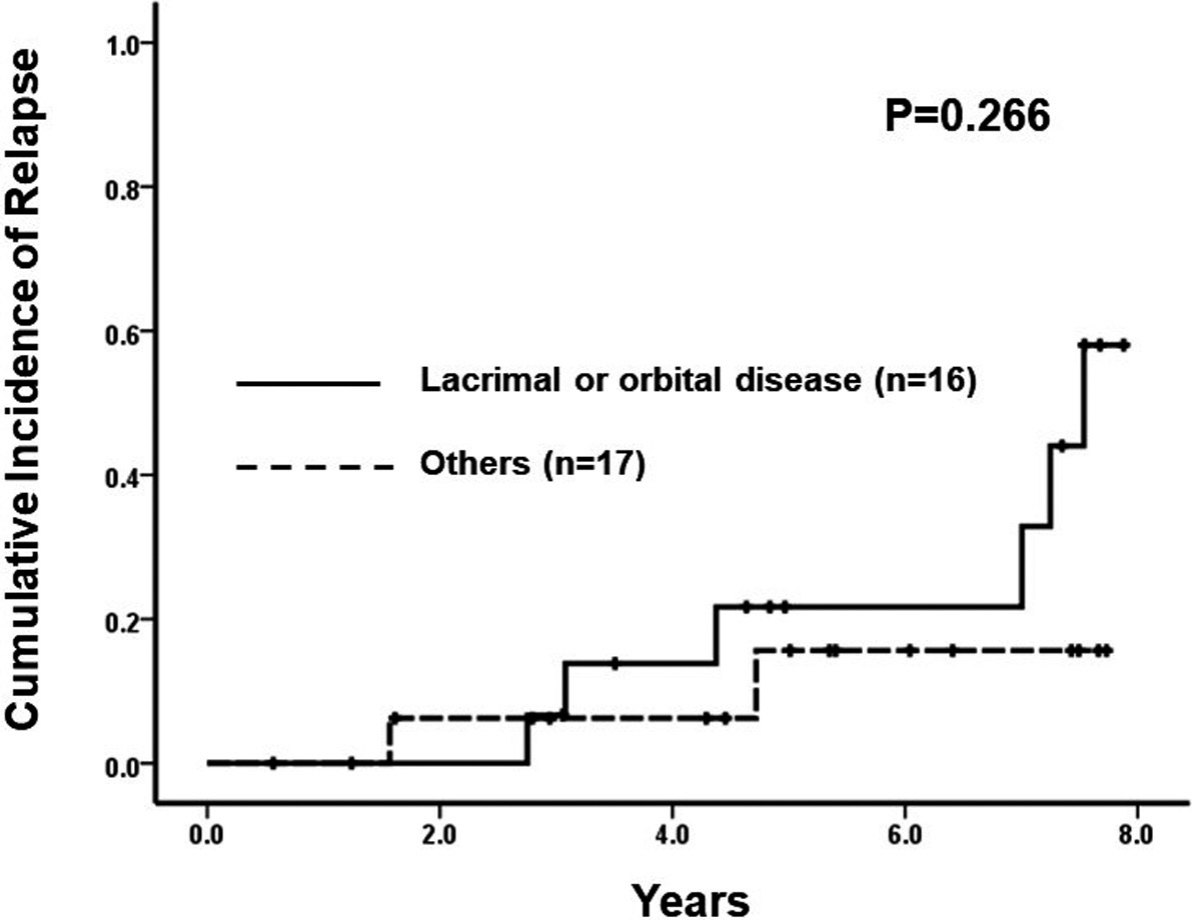
Comparison of cumulative incidence of relapse in patients with lacrimal or orbital disease and in patients with diseases at other locations

Based on statistical analysis, lacrimal and orbital diseases showed a higher progression risk than other diseases (Figure 2; HR: 2.5, 95% CI: 0.498–12.500, *p* = 0.25). However, the multivariate analysis did not reveal any independent prognostic factors (Table 4).

**TABLE 4:** Statistical analysis of factors affecting the cumulative incidence of relapse or progression

Variables	Univariate		Multivariable
	HR (95% CI)	*p* value	*p* value
Age (≥49 vs. <49)	0.354 (0.083–1.508)	0.160	0.279
Sex (Female vs. male)	1.137 (0.270–4.781)	0.861	0.971
LDH (elevated vs. normal)	0.040 (0.000–430.175)	0.498	0.968
Laterality (bilateral vs. unilateral)	0.769 (0.379–1.558)	0.465	0.121
TNM (>T1 vs T1)	0.905 (0.181–4.523)	0.904	0.962
Location (lacrimal and orbital vs. others)	2.495 (0.498–12.500)	0.266	0.954
Time to CR (>4.3 vs. ≤4.3 months)	1.181 (0.277–5.031)	0.822	0.406

Abbreviations: CR, complete response; LDH, lactic dehydrogenase; TNM, tumor, node, and metastasis.

## 4 DISCUSSION

The present study investigated the long‐term clinical outcome in patients with limited‐stage OAML and poor prognostic location after receiving R‐CVP chemoimmunotherapy as a frontline treatment. Although the previous phase 2 study showed that the response rate and short‐term effect of this regimen were remarkable (ORR: 100%, CR: 93.9%, and PFS: 90.3% at 4 years), the present extended observational study revealed that this regimen was not able to maintain sufficient disease control for a long‐term. Although the OS was 100%, the PFS was only 81.1% at 5 years and 55.3% at 8 years. The estimated cumulative incidence of relapse at 8 years was 44.8%. The relapse mostly occurred at more than 4 years after treatment. It was difficult to compare outcomes of radiotherapy and R‐CVP treatment as frontline therapies for this group of patients. Given that prior studies on radiotherapy have reported that the relapse incidence is 40%–50% at 5–8 years for nonconjunctival diseases,^10^, ^12^ the risk of relapse is likely to be similar between the two treatments.

Prior studies evaluating efficacies of different rituximab‐combined chemoimmunotherapy regimens in the treatment of patients with stages I–IV mucosa‐associated lymphoid tissue (MALT) lymphoma have demonstrated promising results.^14^, ^15^, ^21^ However, follow‐up periods in most of those studies were relatively short to accurately observe long‐term outcomes for this indolent disease with a high tendency to show delayed or late relapse. The present study recommends a long‐term observation period to evaluate treatment outcomes of OAML patients even with the limited stage of the disease.

Because of its small sample size, statistical analysis did not reveal any significant prognostic factors for relapse. However, a higher relapse risk was observed in patients with lacrimal or orbital disease than in those with conjunctival or eyelid disease. Of note, in lacrimal disease, the relapse occurred at a different site from that of the primary tumor. Similarly, a previous study has reported that lacrimal and orbital location diseases show poor responses to radiotherapy,^10^ suggesting that tumor location is an important predictor for both radiotherapy and chemoimmunotherapy outcomes. Elevated LDH and old age as prognostic factors for EMZL based on an EMZL‐specific prognostic index were not significant prognostic indicators in this study. In addition, bilaterality, time to CR achievement, and TNM stage had no significant impact on treatment outcomes in the present study.

Previous studies have reported that relapse after radiotherapy is predominately found in non‐irradiated areas.^9^ In the previous phase 2 study, we hypothesized that systemic R‐CVP chemoimmunotherapy could lower the risk of relapse while avoiding radiotoxicity of eyes. However, the present long‐term study showed a relatively high risk of regional relapse at the original tumor site after systemic R‐CVP chemoimmunotherapy. All incidences of relapse occurred at the same location as the original tumor except in lacrimal disease, suggesting that chemoimmunotherapy is likely to be inferior to radiation in the treatment of local disease but superior in the treatment of distant diseases. This study also showed that lacrimal disease had a poor prognosis and an increased risk of distant relapse after both chemoimmunotherapy and radiotherapy.

Because most treatment guidelines recommend radiotherapy as the frontline treatment for limited‐stage OAML regardless of unfavorable prognostic factors for radiotherapy, including the location of OAML tumor,^3^, ^25^ there have been very few clinical trials regarding chemoimmunotherapy for patients with limited‐stage OAML. This study provided long‐term treatment outcomes of chemoimmunotherapy in patients who had a high risk of relapse and significant radiotoxicity when they were treated with radiotherapy. Based on our findings, other therapeutic strategies should be considered for longer disease‐free survival of limited‐stage OAML patients with adverse prognostic factors. Sequential or combinational treatment with radiation and chemoimmunotherapy to enhance the control of regional disease and reduce the risk of distant relapse can be considered for high‐risk patients. The optimal radiation dose for treatment of limited‐stage OAML with high‐risk features needs to be established. Recent studies using low‐ or ultra‐low dose radiation have shown promising results of local disease control with few ocular complications, although their long‐term data for high‐risk patients are limited.^26^, ^27^, ^28^ Therefore, to minimize radiotoxicity, low‐dose or ultra‐dose radiation can be incorporated in this treatment setting. Importantly, clinical trials for this strategy should have a long‐term observation period.

## CONFLICT OF INTEREST

Roche Pharmaceuticals generously provided rituximab, but had no other roles in this study. We have no other conflicts of interest to disclose.

## AUTHORS CONTRIBUTION

Sung‐Yong Kim wrote the manuscript and contributed to conceptualization, visualization, data curation, and formal analysis. Won‐Sik Lee, Sung Yong Oh, Deok‐Hwan Yang, Hyo Jung Kim, and Seong Kyu Park contributed to the investigation and reviewed the draft. Jae Wook Yang and Suk‐Woo Yang contributed to data interpretation and data analysis. Seok‐Goo Cho contributed to conceptualization, methodology, project administration, visualization, and supervision of the study and reviewed the manuscript.

## ETHICS

All relevant ethical guidelines were followed in the conduct of this study. This study was approved by the Institutional Review Boards of all participating centers. All patients provided written informed consent.

## Data Availability

The data that support the findings of this study are available from the corresponding author upon request.
